# SLPI overexpression in hMSCs could be implicated in the HSC gene expression profile in AML

**DOI:** 10.1038/s41598-024-66400-7

**Published:** 2024-07-05

**Authors:** Pedro L. Azevedo, Simone Maradei, Ricardo de Sá Bigni, Jordana Santos Ramires Aragao, Eliana Abdelhay, Renata Binato

**Affiliations:** 1 Stem Cell Laboratory, Lab. de Células-Tronco (LCT) Centro, National Cancer Institute (INCA), Praça da Cruz Vermelha 23, 6° andar, Ala C, Rio de Janeiro, RJ CEP: 20230-130 Brazil; 2 Bone Marrow Transplantation Unit, National Cancer Institute (INCA), Rio de Janeiro, RJ Brazil; 3 Haematology Service, National Cancer Institute (INCA), Rio de Janeiro, RJ Brazil

**Keywords:** Cancer microenvironment, Mesenchymal stem cells, Cancer, Molecular biology, Cancer stem cells, Haematological cancer, Leukaemia, Gene expression analysis, Microarray analysis

## Abstract

Acute myeloid leukaemia (AML) is a severe haematological neoplasm that originates from the transformation of haematopoietic stem cells (HSCs) into leukaemic stem cells (LSCs). The bone marrow (BM) microenvironment, particularly that of mesenchymal stromal cells (hMSCs), plays a crucial role in the maintenance of HSCs. In this context, we explored whether alterations in the secretome of hMSCs derived from AML patients (hMSC-AML) could impact HSC gene expression. Proteomic analysis revealed that the secretome of coculture assays with hMSC-AMLs and HSC from healthy donor is altered, with increased levels of secretory leukocyte protease inhibitor (SLPI), a protein associated with important processes for maintenance of the haematopoietic niche that has already been described to be altered in several tumours. Increased SLPI expression was also observed in the BM plasma of AML patients. Transcriptome analysis of HSCs cocultured with hMSC-AML in comparison with HSCs cocultured with hMSC-HD revealed altered expression of SLPI target genes associated with the cell cycle, proliferation, and apoptosis. Important changes were identified, such as increased expression levels of *CCNA2, CCNE2, CCND2, CD133* and *CDK1* and decreased levels of *CDKN2A* and *IGFBP3,* among others. Overall, these findings suggest that the altered secretome of coculture assays with hMSC-AMLs and HSC from healthy donor, particularly increased SLPI expression, can contribute to gene expression changes in HSCs, potentially influencing important molecular mechanisms related to AML development and progression.

## Introduction

Acute myeloid leukaemia (AML), the most common and severe haematological neoplasm in adults, is a disease characterized by blockade of haematopoietic stem cell (HSC) differentiation and the accumulation of blasts. Although it is considered highly heterogeneous, AML is known to have a unique origin from leukaemic stem cells (LSCs). The onset of AML and the transformation of HSCs could be associated with intrinsic factors, such as genetic alterations in HSCs, and extrinsic factors, including alterations in the bone marrow (BM) microenvironment. Several studies highlight that the crucial role of the dynamic interaction between HSCs and the BM niche is of paramount relevance in the pathogenesis of AML and the origination of LSCs^1–3^.

The haematopoietic niche is extremely complex and dynamic and has a coordinated signalling network^4,5^. Their components include proteins, metabolites, soluble factors, cytokines, and chemokines that mediate important functions in haematopoietic cells. Alterations in the signalling of this BM niche are associated with haematopoietic insufficiency^6^.

Among the cell populations in the BM, mesenchymal stromal cells (hMSCs) have emerged as key regulators of haematopoiesis. hMSCs constitute a nonhaematopoietic cell population that provides structural support by colocating closely with HSCs and regulating their homeostasis through the secretion of proteins essential for controlling the self-renewal, proliferation and differentiation of HSCs and their progenitors^6–8^. Furthermore, hMSCs play a fundamental role in protecting HSCs from stress signals, such as oxidative stress and inflammation, and are also responsible for generating osteoblasts, which provide essential signals for the maintenance of HSCs^9^.

In addition to playing an important role in the control of normal haematopoiesis, hMSCs have been linked to the leukaemic transformation process. hMSCs derived from AML patients (hMSC-AML) exhibit genetic, molecular and functional alterations that may contribute to leukaemogenesis and, in turn, generate an altered niche capable of supporting myeloid neoplasms. However, the contribution of hMSC-AML signalling to the onset of AML is still poorly understood^5,10,11^.

Therefore, in this work, we aimed to evaluate whether hMSC-AMLs are capable of altering the gene expression of HSCs from healthy donors. For this purpose, we performed coculture assays and identified differentially expressed proteins secreted from hMSC-AML cells; among them, SLPI expression was increased in the secretome of hMSC-AML cocultured with HSCs in comparison with hMSC-HD cocultured with HSCs. This increased expression was also confirmed in the plasma of BM derived from AML patients. SLPI is involved in the regulation of cell cycle genes. To verify whether SLPI targets were altered in HSCs, we performed transcriptome analysis of HSCs after coculture assays. Our findings suggest that increased expression of the SLPI protein secreted by hMSC-AML may alter the expression of genes in HSCs from healthy donors and may be related to AML development.

## Results

### Characterization of hMSC and HSC cultures

**Figure 1:**
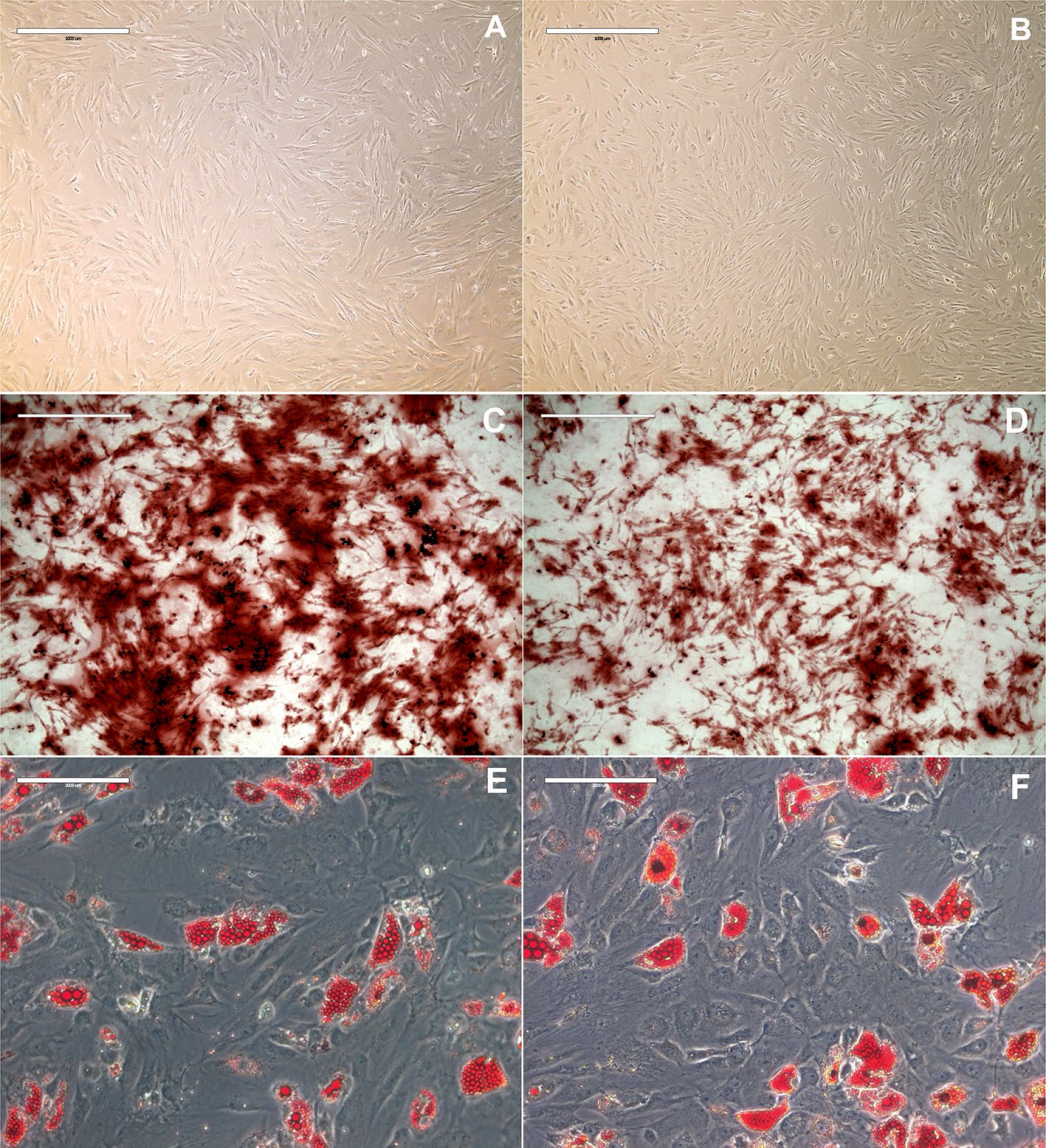
hMSC multipotency capacity. (**A**,**B**) Undifferentiated hMSC-HD and hMSC-AML, respectively (×100 magnification). (**C**,**D**) Osteogenic differentiation of hMSC-HDs and hMSC-AMLs, respectively. Calcium deposition, as determined by Alizarin Red staining, indicated cell differentiation (×100 magnification). (**E**,**F**) Adipogenic differentiation of hMSC-HDs and hMSC-AMLs, respectively. The accumulation of neutral lipid vacuoles stained with Oil Red O indicates cell differentiation (×200 magnification). *hMSC-HD* mesenchymal stromal cells derived from healthy donors, *hMSC-AML* mesenchymal stromal cells derived from AML patients.

To confirm the multipotentiality of the hMSCs used in this work, experiments were performed in accordance with the minimal criteria for defining multipotent mesenchymal stromal cells as defined by the International Society for Cellular Therapy (ISTC). For this purpose, we induced hMSCs from healthy donors (hMSC-HDs) and hMSCs from patients with AML (hMSC-AML) at passage 3 to differentiate into adipogenic and osteogenic cells in vitro to verify the multipotent differentiation capacity of the hMSCs. Undifferentiated hMSC-HDs and hMSC-AMLs were used as controls (Fig. 1A,B). Our results showed that both cultures were able to differentiate, indicating the preservation of their multipotent capacity according to the criteria of the ISTC (Fig. 1C–F). We observed a reduction in the osteogenic differentiation potential of hMSCs-AML in vitro (Fig. 1C,D), consistent with our previously reported findings^12^.

**Figure 2:**
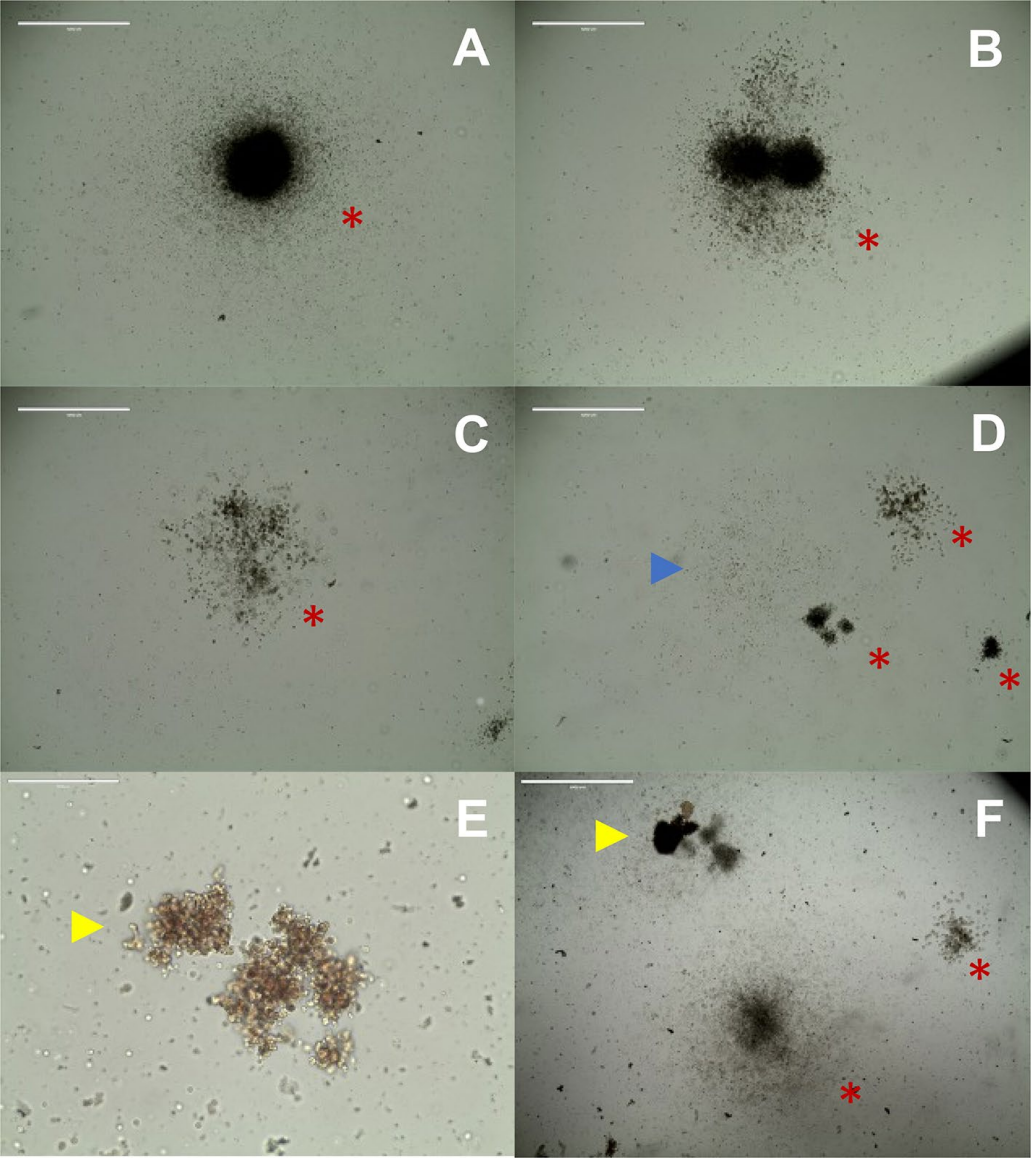
Representative images of colonies formed after the CFC assay of HSCs derived from healthy donors. (**A**–**D**,**F**) Granulocyte and macrophage colony-forming units (CFU-GM), highlighted with a red asterisk, (**D**) macrophage colony-forming units (CFU-M), highlighted with a blue arrow, and (**E**,**F**) Erythroid Colony-forming Units, highlighted with a yellow arrow, were observed from HSCs derived from healthy donors.

We also verified that all HSCs obtained after enrichment with BM mononuclear cells from healthy donors were viable and capable of forming colonies of different haematopoietic cell types. As shown in Fig. 2, after 14 days of culture, it was possible to observe the formation of CFU-GM (granulocyte and macrophage colony forming units) and CFU-M (macrophage colony forming units) colonies. Therefore, we confirmed that HSCs derived from the BM of healthy donors maintained their ability to give rise to normal myeloid progenitors.

### Secretome analysis revealed increased expression of the SLPI protein in coculture from AML condition in comparison with coculture from Healthy condition

Based on the importance of hMSC signalling for the regulation of HSCs in the haematopoietic niche and because, until recently, the exact contribution of molecular alterations in hMSC to the pathogenesis of AML has been poorly understood, evaluating whether there is some difference in the secretome of AML condition when compare with Healthy condition, and its impact on the regulation of HSCs has become important. For this purpose, we initially performed assays on cocultures composed of: 1. HSCs from a healthy donor with hMSC-AML (AML condition) or 2. HSCs from the same healthy donor with hMSC-HD (Healthy condition); after 72 h, the supernatants were collected for proteomic analysis.

The results showed that the secretome of hMSCs in the AML condition was altered. When we compared the protein expression profiles of the supernatants of the AML condition with those of the Healthy condition, we identified 15 differentially expressed proteins, among which 11 proteins were exclusive to the Healthy condition (absent in AML condition), 3 proteins were exclusive to the AML condition (present only in AML condition), and one protein with increased expression was exclusive to the AML condition. The proteins identified in this analysis are described in Table 1.

**Table 1:** List of the 15 differentially expressed proteins identified in the supernatant of coculture assays from AML condition compared with Healthy condition.

Description	Protein expression level (AML condition vs. healthy condition)
Antileukoproteinase (SLPI)	Present
Na(+)/H(+) exchange regulatory cofactor (NHE-RF1)	Present
Homeobox protein (MSX2)	Present
Serum albumin (ALB)	Present (*Fold-Change*: 1,62)
Kidney-associated antigen 1 (KAAG1)	Absent
TP53-regulated inhibitor of apoptosis 1 (TRIAP1)	Absent
Zinc-alpha-2-glycoprotein (AZGP1)	Absent
Transthyretin (TTR)	Absent
Alpha-2-HS-glycoprotein (AHSG)	Absent
Alpha-1-acid glycoprotein 1 (ORM1)	Absent
Beta-2-glycoprotein 1 (APOH)	Absent
Vitamin d-binding protein (GC)	Absent
Short stature homeobox protein 2 (SHOX2)	Absent
Killer cell immunoglobulin-like receptor 3DS1 (KIR3DS1)	Absent
Haptoglobin-related protein (HPR)	Absent

To identify the biological processes related to these differentially expressed proteins, we performed in silico analysis using MetaCore™ software (GeneGO, Inc., USA). Among the biological processes associated with the differentially expressed proteins, we highlighted the processes of bone development and remodelling, proteolysis, signal transduction, inflammation, cell adhesion and immune response (Table 2).

**Table 2:** Biological processes related to the differentially expressed proteins.

Process	Protein expression level (aml condition vs. healthy condition)	
	Present	Absent
Development: Ossification and bone remodeling	MSX2	AHSG
Proteolysis	SLPI	
Signal transduction: BMP and GDF signaling	MSX2	
Cardiac development	MSX2	
Inflammation		ORM1
Cell adhesion		APOH
Immune response		AZGP1

Interestingly, among the identified differentially expressed proteins, we highlighted the increased expression of the secreted protein SLPI in AML. SLPI is related to important processes involved in the maintenance of the haematopoietic niche, such as the homeostasis system, haematopoietic system, regulation of proliferation, cell cycle progression and apoptosis. The SLPI has already been shown to be altered in several tumours, including lung, pancreas, gastric, breast and ovarian cancer, and is involved in tumour progression^13^. However, in AML, the role of the SLPI protein has not been determined.

Furthermore, the observed increased abundance of SLPI protein in coculture supernatants correlated with previously observed increased SLPI mRNA expression in AML patient-derived hMSCs^14^, consistent with the increase in this protein in the hMSC-AML secretome.

These findings indicate an altered secretome profile in hMSC-AML, and these differentially expressed proteins could be related to changes in biological processes crucial for the maintenance of HSCs.

### SLPI protein expression is increased in bone marrow plasma from patients with AML

**Figure 3:**
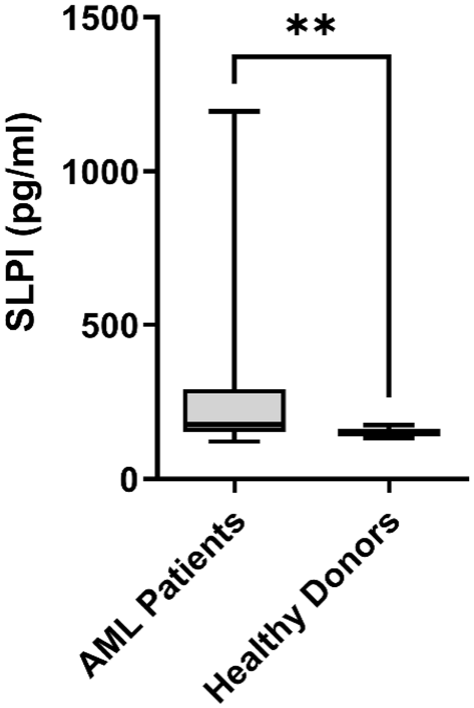
The expression of the secreted protein SLPI is increased in bone marrow plasma from AML patients. The results obtained after ELISAs showed an increase in SLPI protein expression in the bone marrow plasma of patients with AML (n = 26) compared to that in the bone marrow plasma of donors (n = 16). **p < 0.05. *AML* acute myeloid leukaemia.

To verify whether the differences in protein expression found in the proteomic assays from the coculture supernatants were also found in the haematopoietic niche, we isolated BM plasma from a cohort of patients with AML of different prognostic risks and from healthy donors and measured the concentration of the SLPI protein through ELISA. In this analysis, we used 26 plasma samples from patients with AML and compared them with the plasma from 16 healthy donors. ELISA revealed a significant increase in SLPI expression (Fig. 3) in the BM plasma of patients with AML compared to that in the BM plasma of donors.

These findings corroborated the results obtained for the supernatant secretome. Changes in SLPI expression could be related to changes in the haematopoietic niche and could be associated with important biological processes in the maintenance of HSCs.

### Genes regulated by SLPI are altered in HSCs after interaction with hMSC-AML

It has already been reported that secreted SLPI can rapidly enter cells, localize to the cytoplasm and nucleus, and regulate genes related to proliferation, apoptosis and the cell cycle, processes that are frequently altered in cancer^15,16^. In HSCs, SLPI was shown to be capable of regulating the cell cycle through changes in the expression of genes such as *C-MYC*, *CCNE2* and *CDC6*^17^.

**Figure 4:**
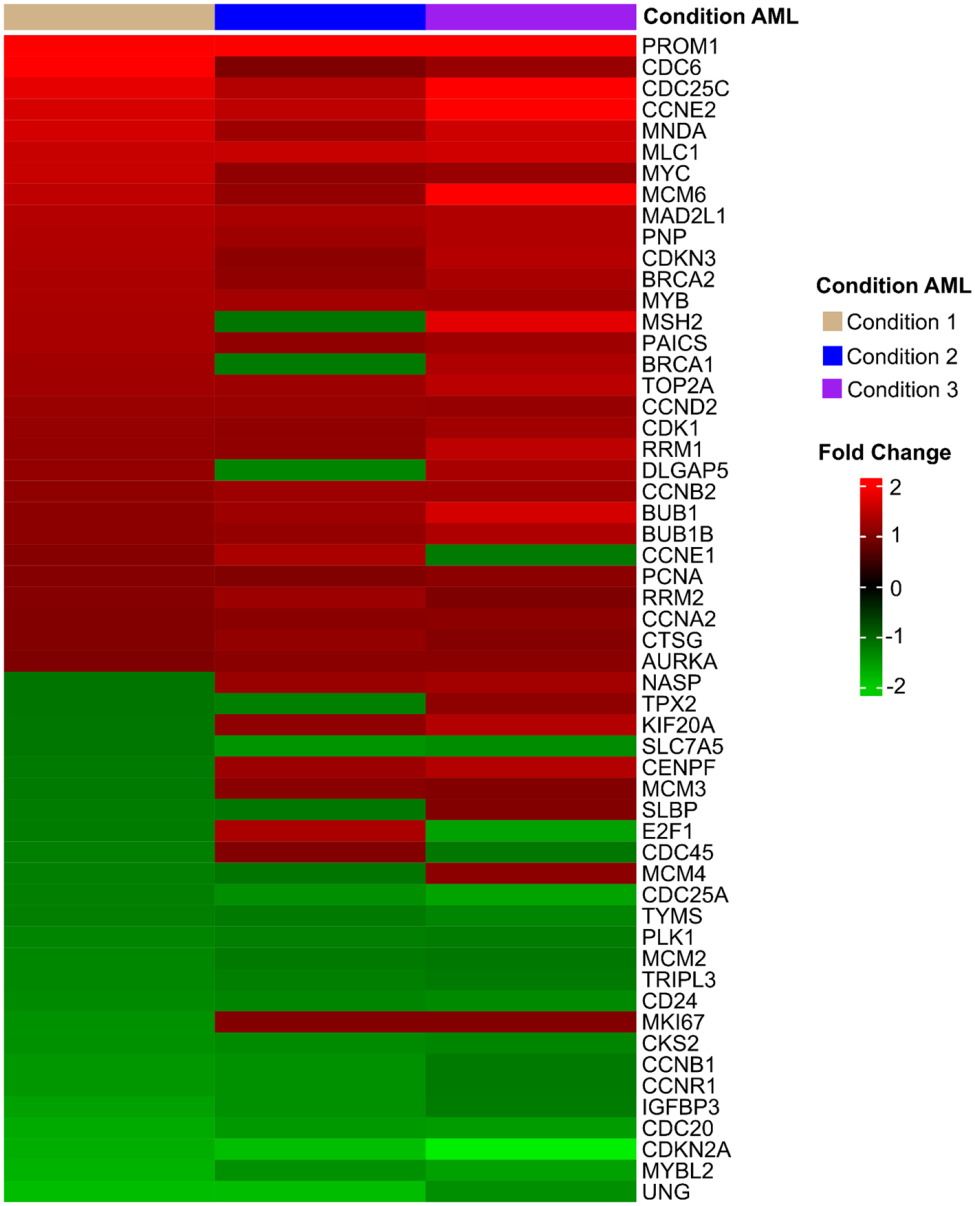
Gene expression profile of SLPI targets associated with the cell cycle are altered in donor-derived HSCs from AML condition in comparison with Healthy condition. Heatmap of SLPI target genes in donor-derived HSCs from AML condition compared to those from Healthy condition. The expression level of each gene is depicted according to the color scale shown on the right. Red and green indicate that the expression levels increased and decreased, respectively, when compared with those in the donor-derived HSCs from AML condition and with Healthy condition. Each row represents a gene, and each column represents the AML condition of the coculture assay. The heatmap was generated using the ComplexHeatmap package (version 2.18.0; ComplexHeatmap, RRID:SCR_017270) in the R software (version 4.3.2; https://www.r-project.org/, accessed on 5 January 2024).

To evaluate whether alterations in the SLPI protein could alter HSC gene expression after coculture, we sought to assess by transcriptomic analysis whether the genes described altered by SLPI were altered in our donor-derived HSCs obtained after interaction with hMSC-AML (AML condition) and hMSC-HD (Healthy condition). These results are described in Fig. 4.

Among the altered genes related to the cell cycle, *MYC*, *Cyclin A2* (*CCNA2*), *Cyclin E2* (*CCNE2*), *Cyclin D2* (*CCND2*), *CD133* (*PROM1*) and *Cyclin-dependent kinase* (*CDK1*) were more highly expressed in the HSCs from AML condition than in those of the Healthy condition (Fig. 4). *MYC* is the most prevalent oncogenic transcription factor involved in many cancers, including AML^18^. High *CCNE2* expression has already been associated with increased genomic instability, which is a key feature of cancer^19^. *CDK1* is a protein essential for cell cycle progression^20^. Furthermore, the *CDKN2A* gene, a cyclin-dependent kinase 2A inhibitor capable of regulating the cell cycle and exhibiting tumour suppressor activity, was found to be downregulated in HSCs from patients with AML. Taken together, our results suggest that increased expression of the SLPI protein can alter the expression of genes associated with the cell cycle in HSCs.

In addition to affecting genes related to the cell cycle, SLPI is also capable of regulating cell proliferation and apoptosis through the downregulation of antiproliferative factors, such as insulin-like growth factor binding protein 3 (*IGFBP3*)^16^. In our study, compared with that in Healthy condition, IGFBP3 in HSCs obtained after coculture assays under AML condition was lower. It has also been described that SLPI regulates HSC apoptosis^17^.

Our results showed that the expression profiles of most of the genes altered by SLPI in HSCs^17^ also changed under our AML conditions after coculture assays. Therefore, SLPI could play an important role in altering the cell cycle, proliferation, and apoptosis processes, and consequently, elevated SLPI expression is associated with the initiation and/or progression of AML.

## Discussion

Although it is considered extremely heterogeneous, AML has a unique origin from the transformation of HSCs into LSCs^1,3^. In recent years, it has been demonstrated that the BM niche plays an active role in myeloid leukaemic transformation and not only plays a passive role. Changes in this niche are believed to contribute to the initiation and progression of leukaemia in a model of malignant transformation guided by the haematopoietic niche^7^.

Several studies have described changes in hMSC-AML that culminate in haematopoietic insufficiency and the development of AML^6,14,21,22^. Therefore, this work focused on understanding the possible changes in signalling and/or biological processes in hMSC-AML that could be related to changes in HSCs and lead to leukaemic transformation and the onset of AML.

Initially, we determined that the hMSC-AML secretome is altered. After proteomic analysis of the supernatant from coculture assays, 15 differentially expressed proteins, including transcription factor, cytoplasmic, structural and secreted proteins, were identified. The presence of transcription factors among the identified proteins in the supernatant corroborates the literature evidence since it has already been established that, in addition to signalling through secreted proteins, hMSCs also signal from vesicles or exosomes. Therefore, hMSCs are capable of releasing these transcription factors into the extracellular environment^23^.

Given our focus on hMSC-AML-secreted proteins that could be involved in the transformation of HSCs into LSCs, we highlighted the increase in the SLPI protein in the supernatant of the AML condition compared to that in Healthy condition.

It is important highlighting that, all HSCs used in the AML condition were obtained from the same healthy donor, which were subjected to co-culture assays, under the influence of MSCs derived from different AML patients. Therefore, the only difference in the AML condition was in AML-MSC signaling, where we identified an increase in SLPI levels, potentially capable of modulating HSC gene expression.

A significant increase in the expression of SLPI was also observed in the bone marrow plasma from AML patients, corroborating the results found in the secretome. Furthermore, we detected an increase in SLPI expression in the transcriptome of isolated hMSC-AML cells compared to that in the transcriptome of hMSC-HD cells, suggesting that the increase in SLPI expression in the haematopoietic niche may be attributed to hMSC-AML signalling.

SLPI has already been described for its cancer-promoting ability, and its increased expression has been found in several tumours, including lung, pancreatic, gastric, breast and ovarian cancer, related to tumour progression^13^. Due to its presence in easily accessible fluids, such as saliva, blood and mucus, SLPI has been widely investigated as a potential diagnostic and prognostic biomarker for cancer^23^.

SLPI is expressed by a variety of cells, including granulocytes, monocytes/macrophages, and epithelial cells, and has always been considered a protease inhibitor protein. However, in recent years it has been described that its biological function is more complex. Research into this protein has expanded from basic biochemistry to studies of systemic diseases, and SLPI has been consistently reported to regulate gene expression. Thus, SLPI likely regulates the expression of many other genes, affects a variety of physiological and pathophysiological processes, and may act mainly as a transcriptional regulator^24^.

SLPI can be internalized into the cell and present cytoplasmic and nuclear localization^15^. In the nucleus, it can act as a transcription factor and bind to consensus sites in specific promoter regions in DNA, for example by binding to NF-kB sites in monocytes and inhibiting p65 binding^25^. Thus, it is possible that the increase in SLPI secretion by AML-MSCs could culminate in the regulation of genes in HSCs from healthy donors, and this would represent a new and intriguing avenue of investigation.

In cancer, SLPI has been shown to be associated with cell proliferation, apoptosis, invasion and metastasis^24,26^.

Klimenkova and colleagues observed that the absence of SLPI in HSCs derived from healthy donors is associated with reduced expression of several genes related to the regulation of myeloid differentiation, the cell cycle and proliferation. The authors showed that these cells exhibited cell cycle arrest and elevated apoptosis. To evaluate whether the increased expression of the secreted protein SLPI found in our study could be responsible for the alteration in gene expression described by Kimenkova, we performed transcriptomic analysis on HSCs obtained after coculture assays. Several SLPI targets presented increased expression in HSCs under AML conditions, corroborating the findings in the literature^17^.

Our results showed that important genes that regulate the cell cycle, such as *MYC* and the cyclins *CCNA2, CCNE2, CCND2*, and *CDK1,* were altered and that their expression was increased in HSCs from patients with AML. Cell cycle proteins are commonly altered in cancer and are often ideal targets for immunotherapy. Interestingly, *MYC*, which was also shown to be increased in our analysis, stimulates cell cycle progression and cell proliferation through the regulation of genes related to cell cycle control^27^. Increased expression has also been reported in more than 90% of a cohort of AML patients^18^. Overexpression of *CCNE2*, which was found in our analysis, has already been detected in cells from AML patients^28^. *CDK1* promotes the G2/M and G1/S transitions, as well as G1 cell cycle progression, and is an indicator of malignancy in cancer^20^. Furthermore, *CDKN2A* is one of the most extensively studied tumour suppressor genes and plays a critical role in cell cycle progression, cellular senescence, and apoptosis^29^. Consequently, the suppression of *CDKN2A* induced by SLPI may facilitate accelerated HSC division.

Interestingly, *CD133,* also known as *PROM1*, is expressed at increased levels in acute myeloid leukaemia (AML) HSCs and has been documented as a marker of cancer stem cells (CSCs) in several human neoplasms, and its expression seems to predict unfavourable prognosis^30^, corroborating the hypothesis that increased SLPI may contribute to the leukaemogenesis process.

*IGFBP-3* mediates antiproliferative and proapoptotic effects and acts as a tumour suppressor. It was shown that *IGFBP-3* induces the growth arrest and apoptosis of human myeloid leukaemia cells^31^. We found that the expression of IGFBP-3 decreased in HSCs from patients with AML, demonstrating that SLPI may also play a role in cell proliferation induction and antiapoptotic processes.

In summary, our results showed that the secretome of hMSC-AML is altered, and the increased expression of the secreted SLPI protein may be associated with an altered gene expression profile in HSCs from healthy donors. These altered genes are associated with important biological processes in AML, such as the cell cycle, proliferation, and apoptosis, indicating that this protein could be important for leukaemic transformation in AML. However, whether these changes are related to the process of leukaemic transformation and/or progression of AML remains to be investigated.

## Methods

### Sample collection

BM samples were obtained from patients with AML at diagnosis (without any treatment) and from healthy donors (HDs) registered at the Bone Marrow Transplantation Unit, National Cancer Institute (INCA) (Rio de Janeiro, Brazil). The AML samples were characterized according to the ELN risk classification^32^ (Table 3). The HD samples were used as controls (Table 4). All samples were obtained in accordance with the guidelines of the local Ethics Committee and the Declaration of Helsinki. This study was approved by the INCA Ethics Committee (no. 06281419.0.0000.5274), and all participants signed informed consent forms.

**Table 3:** List of AML patients who participated in this study.

Laboratory code	% Blasts	Genetic abnormality	Risk category*	Sex	Age
003/12	67	47,Xdel(X)(q23)+i(11)(q10)[13]/46,XX[15]	Intermediate	Female	82
007/12	75	46,XX,t(3;13)(q13;p11),t(16;21)(p11,q22)	Intermediate	Female	59
017/12	40	Unknown	Unknown	Female	62
021/13	60	bZIP in-frame mutated CEBPA	Favorable	Female	22
037/13	80	Unknown	Unknown	Male	35
040/13	84	Unknown	Unknown	Male	28
051/14	67	Normal karyotype	Favorable	Male	50
052/14	90	47,XY,+mar[3]/46,XY[12]	Intermediate	Male	35
053/14	90	Unknown	Unknown	Male	32
013/16	47	47,XX,+[8]/47,XX,del(X)(q22),+8[13]/46,XX[1]	Adverse	Female	80
020/16	40	Unknown	Unknown	Male	64
028/16	33	Normal karyotype	Favorable	Female	40
029/16	77	t(9;11)(p21.3;q23.3)/MLLT3::KMT2A	Intermediate	Male	23
031/16	65	Normal karyotype	Favorable	Male	53
033/16	55	Normal karyotype	Favorable	Male	32
035/17	20	Unknown	Unknown	Male	61
036/17	35	47,XX,+21[20]	Intermediate	Male	60
045/17	58	Unknown	Unknown	Male	51
049/17	–	t(15;17)(q24.1;q21.2)/PML::RARA	Intermediate	Female	60
053/17	70	Normal karyotype	Favorable	Female	55
056/17	86	Unknown	Unknown	Female	65
057/17	74	Normal karyotype	Favorable	Female	53
058/17	76	t(15;17)(q24.1;q21.2)/PML::RARA	Intermediate	Female	25
001/18	81	Normal karyotype	Favorable	Male	35
003/18	89	Normal karyotype	Favorable	Female	61
004/18	79	Unknown	Unknown	Female	39
001/19	79	t(2:14)(q22:q31)	Intermediate	Male	20
002/19	–	Unknown	Unknown	Female	53
003/19	63	Unknown	Unknown	Female	67
004/19	21	47,XX,+8[18]/46,XX[7]	Intermediate	Female	35
005/19	66	@82,XX,Ph [17]?	Adverse	Female	39
006/20	58	Normal karyotype	Favorable	Male	73
002/21	25.5	Normal karyotype	Favorable	Male	46
003/21	41.3	inv(16)(p13.1q22) or t(16;16)(p13.1;q22)/CBFB::MYH11	Adverse	Female	21
007/21	42	Normal karyotype	Favorable	Female	76

*2022 ELN risk classification by genetic at initial diagnosis^32^.

**Table 4:** List of healthy donors who participated in this study.

Laboratory code	Sex	Age
DOD 002/16	Female	53
DOD 014/17	Female	26
DOD 015/17	Female	58
DOD 016/17	Female	60
DOD 076/19	Male	39
DOD 078/19	Male	44
DOD 080/19	Male	46
DOD 089/19	Female	36
DOD 090/19	Male	29
DOD 091/19	Female	40
DOD 092/19	Female	32
DOD 093/19	Female	44
DOD 096/19	Female	26
DOD 097/19	Female	47
DOD 098/19	Male	22
DOD 099/19	Male	39
DOD 100/19	Male	34
DOD 101/19	Female	64
DOD 104/19	Male	29
DOD 108/20	Male	29
DOD 109/20	Male	44
DOD 110/20	Male	58
DOD 111/20	Male	32
DOD 112/20	Male	29
DOD 113/20	Female	43
DOD 002/21	Female	52
DOD 005/21	Male	46
DOD 008/21	Male	35
DOD 003/22	Male	60
DOD 005/22	Female	26

### Isolation, culture, and confirmation of hMSCs

hMSCs derived from BM samples from AML patients and HDs were cultured as previously described^33,34^. The cells were maintained at 37 °C in a humidified atmosphere with 5% CO^2^. When the hMSC cultures reached 80% confluence, the hMSCs were removed from the plates by treatment with 0.05% trypsin (Invitrogen™) for 5 min at 37 °C and then replated in another culture flask at a density of 2 × 10^3^ cells/cm^2^ (passage 1). These processes were repeated until passage 3, when the hMSCs were used for all the experiments. To characterize the hMSCs, experiments were performed in accordance with the minimal criteria for defining multipotent hMSCs as defined by the International Society for Cellular Therapy (ISCT)^35^.

### *HSCs (CD34*^+^*cells)*

To obtain enriched HSCs, BM mononuclear cells were isolated by Histopaque-1077 gradient centrifugation (Sigma‒Aldrich, St. Louis, MO, USA). After that, CD34+ cells were enriched by immunomagnetic selection using the EasySep Human Progenitor Cell Enrichment Cocktail (StemCell Technologies, USA) according to the manufacturer’s instructions. Purity and cell viability were determined according to the International Society of Haematotherapy and Graft Engineering (ISHAGE) guidelines^36–38^. In our assays, we used only cells that presented a viability greater than 98%, which was confirmed by negative staining for 7-amino-actinomycin D (7-AAD; BD Biosciences, Franklin Lakes, NJ). Additionally, after enrichment assays, more than 80% of the cells were positive for the CD34 (BD Biosciences, Franklin Lakes, NJ) and CD45 (BD Biosciences, Franklin Lakes, NJ) markers.

### Colony-forming cell (CFC) assay

The capacity of enriched HSCs to generate haematopoietic clonogenic progenitors was evaluated using a methylcellulose‐based colony formation assay. Briefly, the cells were cultured in MethoCult H4434 for the CFC assay according to the manufacturer’s instructions (Stemcell Technologies, Vancouver, Canada). All cells were cultured in 35 mm culture dishes for 14 days at 37 °C with 5% CO_2_ and humidity. After that, the number of colony**-**forming units on the basis of the morphology of the CFCs was scored, and images were acquired via Stem Cell Technologies Inc., Canadá.

### Coculture assays

To perform coculture assays, hMSCs (3 × 10^5^ cells) were seeded in 24-well culture plates and allowed to adhere until 90% confluence. For coculture, the HSCs (2.5 × 10^5^ cells) were placed in the top chamber using a transwell insert (Corning, USA) with a 0.4 µm pore diameter to permit protein movement between chambers. StemPro™-34 SFM culture medium (Thermo Fisher Scientific, USA) was supplemented with stem cell factor (SCF) at 100 ng/ml (BioAlbra, Brazil), fms-like tyrosine 3 (Flt-3) at 50 ng/ml (BioAlbra, Brazil), and interleukin 3 (IL-3) at 100 ng/ml (BioAlbra, Brazil). The cells were cocultured for 72 h at 37 °C and 5% CO^2^ under two conditions:

1. Healthy condition (Coculture with hMSC-HD and HSC from HD).
2. AML condition (Coculture with hMSC-AML and HSC from HD).

The experiments were based on the methodology proposed by Perdomo-Arciniegas and Vernot^39^. After 72 h of incubation, the hMSCs and HSCs were isolated for RNA extraction, and the supernatant was removed and treated with 1 µL/mL protease inhibitor mixture (GE Healthcare, USA).

### Label-free protein quantitation via mass spectrometry

To evaluate the secretome after the coculture assay, we performed proteomic analysis using supernatants from both the Healthy condition and AML condition. First, the samples were concentrated as previously reported^40^ and quantified using the Bradford method^41^. For tryptic digestion, 200 µg of protein were utilized following established procedures^40^. After digestion, yeast alcohol dehydrogenase was added to a final concentration of 0.2 pmol/μL, which was consistent with standardized quantification.

The samples were subjected to nanoscale chromatographic separation (2DnanoLC) using the nanoACQUITY UPLC system from Waters® according to the methods of Pizzatti and colleagues^42^ (with modifications). Mass spectrometry analysis was performed as described previously^43^. The MS profile was adjusted to ensure that the LC–MS data generated at low energy were effectively acquired at 400 to 2000 m/z intervals, ensuring that all m/z values lower than 400 in the LC/MSE were the result of dissociation in the collision cell.

Database searching and protein quantification were performed as previously reported^40^. The ProteinLynx Global Server v.3.0 (PLGS) with Expression^E^ software was used to process the spectra and databank search results. The UniProtKB databank (release 2019_06_HUMAN) was used with manually reviewed annotations. Only the proteins found in all replicates under each condition were subjected to analysis with the PLGS ^Expression^ tool algorithm. The list of differentially expressed proteins was then subjected to analysis using MetaCore™ (GeneGo, Thomson Reuters) and KEGG Mapper.

### ELISA

An enzyme-linked immunosorbent assay (ELISA) was used to evaluate the concentrations of secretory leukocyte peptidase inhibitor (SLPI) in plasma samples collected previously from AML patients (n = 26) and from healthy donors (n = 16) according to the corresponding manufacturer's instructions. SLPI levels were determined using an ELISA kit (ELK Biotechnology, ELK1876), with a detection range of 62.5–4000 pg/mL and a sensitivity of 27.1 pg/mL. The values were read at 450 nm in an ELISA reader, and SLPI concentrations were calculated from specific calibration curves prepared with known standard solutions. Statistical analysis and graphical representations were performed using GraphPad Prism™ software (GraphPad Software, Inc., CA, USA).

### Expression chip array data analysis

Total RNA was extracted from the HSCs obtained after coculture of AML condition and Healthy condition with a RNeasy Mini Kit (QIAGEN, Hilden, Germany) following the manufacturer’s instructions. Next, 100 ng of total RNA was used to synthesize biotinylated cRNA according to the instructions of the GeneChip Whole Transcription sense target labelling assay (Thermo Fisher, Waltham, MS, USA). The biotinylated cRNA was hybridized to a Clariom™ S Array (Thermo Fisher), washed and stained according to the manufacturer’s protocols. The GeneChip arrays were scanned using a GeneChip® Scanner 3000. The data were analysed using Transcriptome Analysis Console (TAC) software version 4.0 (Thermo Fisher) to identify genes differentially expressed in HSCs obtained from coculture assay between AML condition and Healthy condition.

### Statistical analysis

All experiments were carried out in triplicate, and the data are expressed as the mean ± standard error of the mean. The results were compared using an unpaired Mann–Whitney test, and a p value < 0.05 was considered to indicate statistical significance (**p < 0.01). Statistical analysis and graphical representations were performed using GraphPad Prism™ software (GraphPad Software, Inc., CA, USA).

## Data Availability

The gene expression data reported in this article have been deposited in the Gene Expression Omnibus database (Accession No. GSE253556).
